# Modeling diffusion-governed solidification of ternary alloys – Part 1: Coupling solidification kinetics with thermodynamics^☆^

**DOI:** 10.1016/j.commatsci.2013.05.015

**Published:** 2013-11

**Authors:** M. Wu, J. Li, A. Ludwig, A. Kharicha

**Affiliations:** a Christian Doppler Laboratory for Advanced Process Simulation of Solidification and Melting, University of Leoben, A-8700 Leoben, Austria; b Department of Metallurgy, University of Leoben, A-8700 Leoben, Austria

**Keywords:** Microsegregation, Solidification path, Volume average model, Macrosegregation, Thermodynamics

## Abstract

A method incorporating the full diffusion-governed solidification kinetics and the ternary phase diagram into a multiphase volume average solidification model is presented. The motivation to develop such a model is to predict macrosegregation in castings. A key feature of this model, different from most previous ones which usually assume an infinite solute mixing in liquid (e.g. lever rule, Gulliver–Scheil), is that diffusions in both liquid and solid phases are considered. It is known that models with assumption of an infinite liquid mixing lead to erroneous estimation of the solidification path at the initial stage. Here solidification of a ternary alloy (Fe–0.45 wt.%C–1.06 wt.%Mn) is examined. As the two chosen alloy elements (C and Mn) have large differences in the solute partition coefficient, liquidus slope and liquid diffusion coefficient, the solidification path shows differently from those predicted by infinite liquid mixing models. The first part of this two-part investigation evaluates the full diffusion-governed kinetics and its influence on solidification path and microsegregation. Applications of the model for the calculation of solidification and macrosegregation are presented in the accompanying paper [Part 2].

## Highlights

• A full diffusion-governed solidification model for ternary alloys.
• Flow and crystal sedimentation during solidification.
• Bridging microsegregation and macrosegregation.
• Evaluation against available analytical models and experiments.

## 1 Introduction

Most solidification models applicable at the casting process scale are based on the predefined solidification path (the trace of liquid or solid composition and its dependence on the evolution of solid phase). For the modeling purpose, this solidification path is often provided in a form of $f_{\text{s}}-T$ function, i.e. evolution of the solid phase fraction as function of temperature. In order to get this function assumption according to lever rule (thermodynamic equilibrium between liquid and solid phases, and infinite diffusion in both phases) or according to Gulliver–Scheil (thermodynamic equilibrium at the liquid–solid interface, and infinite diffusion in liquid and no diffusion in solid) is made. However, the real solidification path is in fact the outcome of the combined thermodynamics, diffusion-governed growth kinetics, and flow and solute transport. It is known that models with assumption of an infinite liquid mixing lead to erroneous estimation of the solidification path, especially at the initial stage. The solidification path is not pre-determinable.

The recent development of computational thermodynamics (CALPHAD) has allowed considering more phenomena such as cooling rate, back diffusion and coarsening for the description of the phase evolution and solidification path [1], [2], [3], [4], [5], [6], [7]. These considerations are, however, confined to solidification in a small ‘isolated volume’ under a given cooling condition. Inside the ‘isolated volume’, mass and species are conserved, but no exchange with outside is allowed. Again the solidification path determined with CALPHAD method does not meet the need of a system where the macroscopic transport phenomena (melt flow and transport of crystals) are essential. Therefore, Combeau and other researchers proposed a micro–macro (or dual scale) segregation model [8], [9], [10], [11], in which an ‘open volume’, corresponding to a discretized volume element out of the global transport system, was considered. The average composition of the volume element is the outcome of the computed result of the global transport system. It was verified [8] that for a binary alloy (Al–Cu) in which the diffusion coefficient of the solute element in the liquid is 2–3 orders of magnitude larger than that in the solid, it is not necessary to consider finite diffusion kinetics in the liquid. Therefore, the assumption of infinite liquid mixing is valid. However, no further studies on ternary (or multicomponent) alloy systems were performed, in which some alloy elements (e.g. Mn) have large difference in the diffusion coefficient between liquid and solid, while others (e.g. C in steel) have very close diffusion coefficient in liquid and solid.

The most promising method for solving the global transport equations during solidification, taking the multiphase nature into account, is the volume average approach [12], [13], [14], [15], [16], [17], [18]. The idea is to treat the melt and different morphologies of solid (equiaxed and/or columnar) as spatially coupled and interpenetrating continua. Here, the equiaxed and columnar crystals are considered as separate phases owing to their difference in hydrodynamics, although they share the same crystallography. The global conservation equations of mass, momentum, species and enthalpy are solved for each phase. The volume average multiphase approach provides further flexibility for handling the full diffusion-governed solidification kinetics. Unfortunately, most of the multiphase models were implemented for binary alloy systems. There are several models which were applied to ternary [19], [20] or multicomponent [21], [22] alloy systems, but the diffusion-governed solidification kinetics is not explicitly treated in these ternary/multicomponent solidification models.

In previous publications the present authors proposed a method to incorporate the thermodynamics of ternary alloys and liquid diffusion-governed solidification kinetics into a multiphase volume average solidification model [23], [24]. Back diffusion was disregarded. A way to access the thermodynamic data (e.g. Thermo-Calc [1]) through a tabulation and interpolation program ISAT (In Situ Adaptive Tabulation) was suggested. With the ISAT approach it is possible to perform an online call of the thermodynamic data and trace the formation of each individual solid phase (primary, peritectic, eutectic, etc.). As the number of calls of the thermodynamic data is equal to the product of the number of the discretized volume elements, the time steps and the calculation iterations per time step, the calculation becomes exhausting. Therefore, the current model is a modification of the previous model using a linearized phase diagram, and no online call of thermodynamic data is necessary. In addition, the model presented in this paper is extended to consider the back diffusion into the solid. With these modifications, the model can be used to perform casting process simulations with incorporated full diffusion-governed solidification kinetics for ternary alloys at a reasonable computation cost.

## 2 Model description

### 2.1 Eulerian volume average model for mixed columnar-equiaxed solidification

**Fig. 1:**
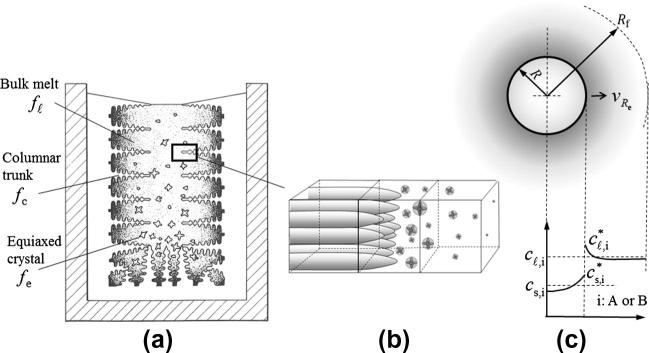
Schematic of (a) the mixed columnar-equiaxed solidification of an ingot, (b) representative volume elements and (c) solute partitioning at the liquid–solid interface and the solute distribution in liquid and solid for the case of non-dendritic crystal growth.

The typical mixed columnar-equiaxed solidification is schematically shown in Fig. 1. The main considerations and assumptions of the model are described as follows:

1. Three phases are involved: primary liquid (ℓ), equiaxed phase (*e*), columnar phase (*c*). They are quantified by their volume fractions: $f_{\ell }$, *f_e_*, *f_c_*.
2. Ideal crystal morphologies are assumed: spheres for equiaxed (globular) grains, and cylinders for columnar (cellular) dendrite trunks. The dendritic morphology can be treated [25], [26], but it is not included in the current model.
3. Both liquid and equiaxed phases are moving phases for which the corresponding momentum conservation equations are solved for the velocity fields ${\overset{\to }{u}}_{\ell }$ and ${\overset{\to }{u}}_{e}$. The columnar phase without motion is assumed to solidify from the mold wall towards the bulk melt.
4. As shown in Fig. 1, after discretization of the casting domain three types of volume elements are distinguished. In the volume element ahead of the columnar tips, only equiaxed (*e*) and liquid (*ℓ*) phases coexist. In the element which includes columnar primary dendrite tips, all three phases coexist. In the element which has already been passed by the columnar primary dendrite tip front, again all three phases are allowed to coexist. In this regard, the position of the columnar primary dendrite tips are explicitly tracked [16], [17].
5. Enthalpy equations for all three phases are solved. Due to the fact that thermal diffusion is much higher than solute diffusion, we assume that only one temperature (*T*) represents each volume element. Therefore, a large inter-phase volume heat transfer coefficient between the phases is applied to balance the temperatures among the phases.
6. Volume-averaged concentrations (*c_ℓ,i_*, *c_e,i_*, *c_c,i_*) in different phases are solved, where *i* = *A* or *B*, indicating different solute elements. At the liquid/solid interface, thermodynamic equilibrium concentrations, $c_{\ell \text{,}i}^{*}\text{,}c_{s\text{,}i}^{*}$, are assumed for the normal cooling condition. Solute partitioning at the interface occurs. The concentration differences ($c_{\ell \text{,}i}^{*}-c_{\ell \text{,}i}$) and ($c_{s\text{,}i}^{*}-c_{s\text{,}i}$) are driving forces for the diffusions in the liquid and solid. The phenomena such as cross diffusion and thermo-migration which are critical for some alloys [27] are currently disregarded. For a very high cooling rate condition (or the diffusion coefficient is small), when the thermodynamic equilibrium condition is violated at the interface, a simple approach is introduced to consider ‘solute trapping’.
7. A continuous 3-parameter (Gaussian) heterogeneous nucleation law is applied in order to model the equiaxed grain nucleation. The transport of grains is also considered. Grain fragments brought into the bulk melt during filling, further fragmentation of dendrites during solidification, and the incorporation of equiaxed grains into the growing columnar phase (consumption of equiaxed phase by the columnar one) are disregarded.
8. The size (*d_e_*), the number density (*n_e_*) of equiaxed grains, and the diameter (*d_c_*) of the columnar trunks are explicitly calculated. In contrast, a constant value for the primary arm spacing ($\lambda_{1}$) of the columnar trunks is used.
9. The densities of the solid and liquid are considered constant and equal. The Boussinesq approach is employed to model thermo-solutal convection, grain sedimentation, and sedimentation-induced melt convection. Feeding flow due to density change in the mushy zone can also be modeled if an open calculation domain is considered (e.g. in continuous casting) [28].

The conservation equations and corresponding solution procedure were presented previously [16], [17]. A key important detail of the model is the calculation of the volume-averaged mass transfer (*M_ℓs_*, kg m^−3^ s^−1^) and species transfer rates ($C_{\ell s}^{i}$, kg m^−3^ s^−1^):

(1) $$M_{\ell \text{s}}=\nu_{R_{s}}\cdot S_{\ell s}\cdot \rho_{s}\cdot \Phi_{\text{imp}}$$

(2) $$C_{\ell s}^{i}=c_{s\text{,}i}^{\text{Intf}}\cdot M_{\ell s}+S_{\ell s}\cdot \rho_{s}\cdot D_{s\text{,}i}\cdot \frac{c_{s\text{,}i}^{\text{Intf}}-c_{s\text{,}i}}{l_{s}}$$

where subscript ‘*s*’ stands for solid phase and superscript or subscript ‘*i*’ stands for the species, *A* or *B*. $\rho_{s}$ is the density and $D_{s\text{,}i}$ the diffusion coefficient of element *i* in the solid. $\Phi_{\text{imp}}$ is a growth surface impingement factor, $S_{\ell s}$ is the surface area concentration (integral of the growth or diffusion surface area per volume, m^−1^), and $l_{s}$ is the diffusion length in the solid (m). Both are estimated according to the spatial arrangement of the crystals as listed in Table 1. $\nu_{R_{s}}$ is the averaged growth velocity (m s^−1^) at the liquid–solid interface and $c_{s\text{,}i}^{\text{Intf}}$ the solid-side interface concentration. Calculations of $\nu_{R_{s}}$ and $c_{s\text{,}i}^{\text{Intf}}$ are described in the following section.

**Table 1:** Spatial arrangement of the crystals and geometrical quantities.

	Equiaxed		Columnar	
	Body-centered cubic	Face-centered cubic	Aligned array	Staggered array
*S_ℓs_*	$n_{e}\cdot \pi d_{e}^{2}$	$n_{e}\cdot \pi d_{e}^{2}$	$\frac{\pi d_{c}}{\lambda_{1}^{2}}$	$\frac{2}{\sqrt{3}}\cdot \frac{\pi d_{c}}{\lambda_{1}^{2}}$
*R_f_*	$\sqrt[{3}]{3/4\pi n_{e}}$	$\sqrt[{3}]{3/4\pi n_{e}}$	$\lambda_{1}/\sqrt{\pi }$	$\sqrt{\sqrt{3}/2\pi }$
*l_ℓ_*	*R_e_*(1 − *R_e_*/*R_f_*)	*R_e_*(1 − *R_e_*/*R_f_*)	*R_c_* ln (*R_f_*/*R_c_*)	*R_c_* ln (*R_f_*/*R_c_*)
*l_s_*	*R_e_*/2	*R_e_*/2	*R_c_*/2	*R_c_*/2
*Φ*_imp_	$\min\left[\frac{f_{\ell }}{1-\pi \sqrt{3}/8}\text{,}1\right]$	$\min\left[\frac{f_{\ell }}{1-\pi \sqrt{2}/6}\text{,}1\right]$	$\min\left[\frac{f_{\ell }}{1-\pi /4}\text{,}1\right]$	$\min\left[\frac{f_{\ell }}{1-\pi /\sqrt{12}}\text{,}1\right]$

### 2.2 Diffusion-governed crystal growth

**Fig. 2:**
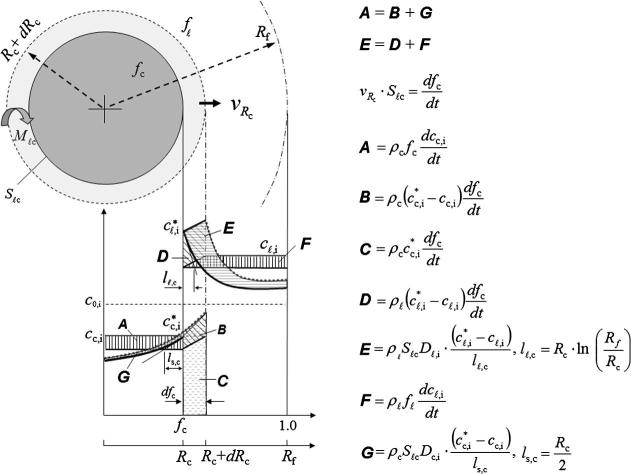
Growth model of a cylindrical columnar trunk and the interfacial species transfer in a cylindrical volume enveloped with a radius *R_f_*. The species increments by different species transfer mechanisms in a certain time step, *dt*, are schematically indicated by the area ***A***–***G***. The formulations in the figure can also be applied to the growth of the spherical equiaxed crystal by replacing the subscript ‘*c*’ with ‘*e*’ but with different diffusion lengths (Section 2.1).

The growth of the cylindrical columnar trunk is schematically shown in Fig. 2. Firstly, the growing cylinder is assumed to be confined in a cylindrical volume enveloped with a radius of *R_f_*, which is determined according to the arrangement of the trunks (aligned or staggered array) and the primary dendrite spacing ($\lambda_{1}$); see Table 1. If the cylindrical volume is considered ‘isolated’, mass and species must conserve, i.e. $d(f_{\ell }\rho_{\ell }c_{\ell \text{,}i}+f_{c}\rho_{c}c_{c\text{,}i})/\mathrm{dt}=0$. With the assumption of constant and equal densities (*ρ_c_* = *ρ_ℓ_*), we may write:

(3a) $$\rho_{c}f_{c}\frac{{\mathrm{dc}}_{c\text{,}i}}{\mathrm{dt}}+\rho_{c}c_{c\text{,}i}\frac{{\mathrm{df}}_{s}}{\mathrm{dt}}+\rho_{\ell }f_{\ell }\frac{{\mathrm{dc}}_{\ell \text{,}i}}{\mathrm{dt}}+\rho_{\ell }c_{\ell \text{,}i}\frac{{\mathrm{df}}_{\ell }}{\mathrm{dt}}=0$$

or with the notation given in Fig. 2:

(3b) $$A+\rho_{c}c_{c\text{,}i}^{*}\frac{{\mathrm{df}}_{c}}{\mathrm{dt}}-B+F-\rho_{\ell }c_{\ell \text{,}i}^{*}\frac{{\mathrm{df}}_{c}}{\mathrm{dt}}+D=0$$

As ***A*** = ***B*** + ***G*** and ***D*** + ***F*** = ***E***, we can write:

(4) $$G+E=\rho_{\ell }(c_{\ell \text{,}i}^{*}-c_{c\text{,}i}^{*})\frac{{\mathrm{df}}_{c}}{\mathrm{dt}}$$

Replacing ***E***, ***G*** and $\frac{{\mathrm{df}}_{c}}{\mathrm{dt}}$ with the expressions given in Fig. 2, we obtain the columnar growth velocity of:

(5) $$\nu_{R_{c}}=\frac{D_{\ell \text{,}i}}{l_{\ell \text{,}c}}\cdot \frac{\left(c_{\ell \text{,}i}^{*}-c_{\ell \text{,}i}\right)}{\left(c_{\ell \text{,}i}^{*}-c_{c\text{,}i}^{*}\right)}+\frac{D_{c\text{,}i}}{l_{s\text{,}c}}\cdot \frac{\left(c_{c\text{,}i}^{*}-c_{c\text{,}i}\right)}{\left(c_{\ell \text{,}i}^{*}-c_{c\text{,}i}^{*}\right)}$$

Using this equation to substitute $\nu_{R_{s}}$ in Eq. (1), the volume-averaged net mass transfer rate for columnar solidification, $M_{\ell c}$, can now be calculated. The diffusion lengths (Table 1), $l_{\ell \text{,}c}=R_{c}\cdot \ln(R_{f}/R_{c})$ and $l_{s\text{,}c}=R_{c}/2$, are estimated from the analytical solution of diffusion fields around/in a cylinder. The liquid diffusion length, $l_{\ell \text{,}c}$, reduces to 0 when $f_{\ell }$ approaches 0, i.e. the impingement of the solute fields of neighboring grains is considered.

Similarly, we can derive the growth velocity for globular equiaxed crystal:

(6) $$\nu_{R_{e}}=\frac{D_{\ell \text{,}i}}{l_{\ell \text{,}e}}\cdot \frac{\left(c_{\ell \text{,}i}^{*}-c_{\ell \text{,}i}\right)}{\left(c_{\ell \text{,}i}^{*}-c_{e\text{,}i}^{*}\right)}+\frac{D_{e\text{,}i}}{l_{s\text{,}e}}\cdot \frac{\left(c_{e\text{,}i}^{*}-c_{e\text{,}i}\right)}{\left(c_{\ell \text{,}i}^{*}-c_{e\text{,}i}^{*}\right)}\text{.}$$

The diffusion lengths (Table 1), $l_{\ell \text{,}e}=R_{e}(1-R_{e}/R_{f})$ and $l_{s\text{,}e}=R_{e}/2$, are estimated from the analytical solution of diffusion fields around/in a sphere. The impingement of the solute fields of neighboring grains is also considered.

The derivation of diffusion lengths, $l_{\ell \text{,}c}$ and $l_{\ell \text{,}e}$, is based on the restriction of the species conservation in the ‘isolated’ cylindrical or spherical volume. We assume that both $l_{\ell \text{,}c}$ and $l_{\ell \text{,}e}$ apply to the case of an ‘open volume element’, where the volume-averaged liquid concentration, $c_{\ell \text{,}i}$, is allowed to be altered by the global species transport.

In Eq. (2), $c_{s\text{,}i}^{\text{Intf}}$ needs to be determined for the interphase species transfer. Solidification and remelting are treated differently. For solidification, two solute partition mechanisms are distinguished. For an alloy element of small solute partition coefficient, *k_i_*, and large liquid diffusion coefficient, *D_ℓ_*_,_*_i_*, when $c_{s\text{,}i}^{*}\leq c_{\ell \text{,}i}$, as shown in Fig. 1c, we assume $c_{s\text{,}i}^{\text{Intf}}$ = $c_{s\text{,}i}^{*}$. This solute partition mechanism operates in most cases, when the cooling rate is small. For an alloy element of large *k*_i_ and small $D_{\ell \text{,}i}$ in a condition of rapid cooling, when $c_{s\text{,}i}^{*}>c_{\ell \text{,}i}$, the above partition mechanism ($c_{s\text{,}i}^{\text{Intf}}=c_{s\text{,}i}^{*}$) would lead to the following problem. As the solid-side interface concentration, $c_{s\text{,}i}^{*}$, becomes larger than the liquid average concentration, $c_{\ell \text{,}i}$, the solute being transferred from liquid to solid due to solidification would be more than the solute which the average liquid phase contains. As a consequence, the liquid average concentration (*c_ℓ_*_,_*_i_*) would gradually decrease until the solute in the liquid phase is fully consumed (*c_ℓ_*_,_*_i_* → 0). This is not true in reality for an alloy of *k_i_* < 1. It is known that at a very high cooling rate (or when the diffusion coefficient of the solute element is very small), the thermodynamic equilibrium condition at the liquid/solid interface could be violated and a solute trapping phenomenon would occur [29], [30]. The partition coefficient is no longer constant, but falls in a range between *k_i_* and 1, dependent on the growth velocity. In the current model, the growth velocity dependent partition coefficient is not considered. Therefore, a simple numerical approach is introduced. When $c_{s\text{,}i}^{*}>c_{\ell \text{,}i}$, we assume $c_{s\text{,}i}^{\text{Intf}}$ = $c_{\ell \text{,}i}$. This means that the solute in the liquid with the average concentration of $c_{\ell \text{,}i}$ is assumed to be fully trapped in the solid phase. This treatment is crude, but it supports one general experimental fact: that segregation phenomenon disappears in a low-diffusive alloy under rapid solidification.

Similarly, two cases are distinguished for remelting. For an alloy element with a small solid diffusion coefficient, $D_{s\text{,}i}$, we assume a crude treatment where $c_{s\text{,}i}^{\text{Intf}}=c_{s\text{,}i}$ [12], [13], [14]. The changes in the average solid concentration during remelting actually depend on the concentration profile ‘frozen’ in the solid during an earlier period. A correct model should be able to record the evolution history of this concentration profile during solidification by ‘remembering’ the interface solid concentrations at all times during remelting. This is not possible for the current model, especially when the transport of the crystals is also considered. For an alloy element with a large solid diffusion coefficient, $D_{s\text{,}i}$, a thermodynamic equilibrium approach can be used by considering $c_{s\text{,}i}^{\text{Intf}}=c_{s\text{,}i}^{*}$ [31].

### 2.3 Coupling of growth kinetics with thermodynamics

**Fig. 3:**
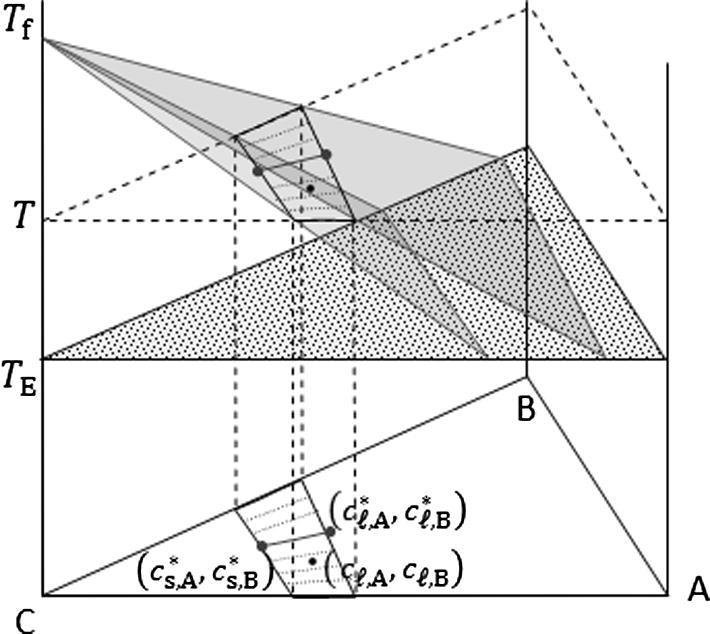
C-rich corner of an A–B–C linearized phase diagram.

Fig. 3 schematically shows the C-rich corner of an A–B–C linearized phase diagram. The expression for the liquidus surface is:

(7) $$T=T_{f}+m_{L\text{,}A}c_{\ell \text{,}A}^{*}+m_{L\text{,}B}c_{\ell \text{,}B}^{*}$$

where $m_{L\text{,}A}$ and $m_{L\text{,}B}$, corresponding to $\partial T/\partial c_{\ell \text{,}A}^{*}$ and $\partial T/\partial c_{\ell \text{,}B}^{*}$, are the slope of the equilibrium liquidus surface in respect to the corresponding solute element. $T_{f}$ is the melting point of pure metal C. Tie lines are defined by:

(8) $$c_{s\text{,}A}^{*}=k_{A}\cdot c_{\ell \text{,}A}^{*}$$

(9) $$c_{s\text{,}B}^{*}=k_{B}\cdot c_{\ell \text{,}B}^{*}$$

where $k_{A}$ and $k_{B}$ are the partition coefficients. In the vicinity of the C-rich corner, *m_L_*_,_*_A_*, *m_L_*_,_*_B_*, *k_A_* and $k_{B}$ are assumed to be constant.

Taking the growth of the columnar trunk as an example, the growth velocities derived according to the diffusion of solute element *A* and *B* must be equal, i.e. ${\left(\nu_{R_{c}}\right|}_{i=A}={\left(\nu_{R_{c}}\right|}_{i=B}$,

(10) $$\frac{D_{\ell \text{,}A}}{l_{\ell \text{,}c}}\cdot \frac{\left(c_{\ell \text{,}A}^{*}-c_{\ell \text{,}A}\right)}{\left(c_{\ell \text{,}A}^{*}-c_{c\text{,}A}^{*}\right)}+\frac{D_{c\text{,}A}}{l_{s\text{,}c}}\cdot \frac{\left(c_{c\text{,}A}^{*}-c_{c\text{,}A}\right)}{\left(c_{\ell \text{,}A}^{*}-c_{c\text{,}A}^{*}\right)}=\frac{D_{\ell \text{,}B}}{l_{\ell \text{,}c}}\cdot \frac{\left(c_{\ell \text{,}B}^{*}-c_{\ell \text{,}B}\right)}{\left(c_{\ell \text{,}B}^{*}-c_{c\text{,}B}^{*}\right)}+\frac{D_{c\text{,}B}}{l_{s\text{,}c}}\cdot \frac{\left(c_{c\text{,}B}^{*}-c_{c\text{,}B}\right)}{\left(c_{\ell \text{,}B}^{*}-c_{c\text{,}B}^{*}\right)}$$

Eq. (10) serves as the equation of number 4, in addition to Eqs. (7), (8), (9), for solving the four unknowns, $c_{\ell \text{,}A}^{*}$, $c_{\ell \text{,}B}^{*}$, $c_{s\text{,}A}^{*}$ and $c_{s\text{,}A}^{*}$. Eq. (10) is nonlinear. The solution procedure of the non-linear equation system Eqs. (7), (8), (9), (10) is described in Appendix A. The volume-averaged concentration $c_{\ell \text{,}A}$ and $c_{\ell \text{,}B}$, and the temperature *T* are calculation results of global species and energy conservation equations.

For the growth of equiaxed crystals, the same form of Eq. (10) applies but different diffusion lengths, $l_{\ell \text{,}e}$ and $l_{s\text{,}e}$, are used instead of $l_{\ell \text{,}c}$ and $l_{s\text{,}c}$. The diffusion coefficients of the two components, $D_{\ell \text{,}A}$ and $D_{\ell \text{,}B}$, or $D_{c\text{,}A}$ and $D_{c\text{,}B}$, differ but there is no difference between $D_{c\text{,}A}$ and $D_{e\text{,}A}$, or between $D_{c\text{,}B}$ and $D_{e\text{,}B}$.

### 2.4 Coupling strategy

The volume-averaged quantities $(f_{\ell }\text{,}f_{c}\text{,}f_{e}\text{,}{\overset{\to }{u}}_{\ell }\text{,}{\overset{\to }{u}}_{e}\text{,}T\text{,}c_{\ell \text{,}i}\text{,}c_{c\text{,}i}\text{,}c_{e\text{,}i}$) are calculated by means of the global conservation equations [16], [17]. In order to close the conservation equations, a volume-averaged mass transfer rate $M_{\ell c}$ (or $M_{\ell e}$) and volume-averaged species transfer rate $C_{\ell c}^{i}$ (or $C_{\ell e}^{i}$), namely the crystal growth velocity, $\nu_{R_{c}}$ (or $\nu_{R_{e}}$), are required. The growth velocity is a function of thermodynamic equilibrium concentrations ($c_{\ell \text{,}i}^{*}$, $c_{c\text{,}i}^{*}$ or $c_{e\text{,}i}^{*}$), as expressed in Eqs. (5), (6). Therefore, the thermodynamic equilibrium concentrations ($c_{\ell \text{,}i}^{*}$, $c_{c\text{,}i}^{*}$ or $c_{e\text{,}i}^{*}$) firstly have to be solved by Eqs. (7), (8), (9), (10) on the basis of the volume-averaged quantities (*T*, *c_ℓ_*_,_*_i_*, *c_c_*_,_*_i_* or *c_e_*_,_*_i_*) from the last iteration. This coupling strategy differs from the micro-macro model as suggested by Combeau et al. [8], where the specific (averaged) enthalpy and averaged mix concentration of the local volume element are calculated in the macroscopic model and inserted into the microscopic model. In the current model it is the volume-averaged quantities (*T*, *c_ℓ_*_,_*_i_*, *c_c_*_,_*_i_* or *c_e_*_,_*_i_*) which are calculated by means of the macroscopic model. The thermodynamic equilibrium concentrations ($c_{\ell \text{,}i}^{*}\text{,}c_{c\text{,}i}^{*}$ or $c_{e\text{,}i}^{*}$) and the liquid–solid interface growth velocity ($\nu_{R_{e}}$or $\nu_{R_{c}}$) are the outcome of the solution of Eqs. (7), (8), (9), (10).

## 3 Calculated solidification path

As an evaluation effort the model is compared with an analytical solution of the solidification path under the given conditions of infinite liquid mixing [30], [32], [33]:

(11) $$c_{\ell \text{,}i}=c_{0\text{,}i}{\left[1-\left(1-2\alpha_{i}^{\prime }k_{i}\right)f_{e}\right]}^{-\frac{1-k_{i}}{1-2\alpha_{i}^{\prime }k_{i}}}$$

where $\alpha_{i}^{\prime }$ is the modified dimensionless solid-state back-diffusion parameter given by:

(12) $$\alpha_{i}^{\prime }=\alpha_{i}\cdot \left(1-e^{-\frac{1}{\alpha_{i}}}\right)-0.5\cdot e^{-\frac{1}{2\alpha_{i}}}\;\text{with}\;\alpha_{i}=\frac{4D_{s\text{,}i}t_{f}}{l_{s}^{2}}$$

Here, $t_{f}$ is the characteristic solidification time. $l_{s}$ is the characteristic diffusion length, approximated as half of the grain radius, *R_e_*/2_,_ in the case of globular equiaxed solidification.

For the low solid diffusivity (*D_s_*_,_*_i_* → 0), $\alpha_{i}^{\prime }\approx \alpha_{i}\approx 0$, Eq. (11) is reduced to the solution of the Gulliver–Scheil approach; for the large solid diffusivity (*D_s_*_,_*_i_* → ∞), $\alpha_{i}\to \infty$, $\alpha_{i}^{\prime }\approx 0.5$, Eq. (11) is reduced to the solution of the lever rule approach.

The analytical solutions of Gulliver–Scheil and lever rule approaches should be reproducible by the numerical model. To check this, solidification of a Fe–0.45 wt.% C–1.06 wt.% Mn ternary alloy without flow and grain sedimentation is first simulated. In Part 1 of this two-part investigation we consider only one solid phase, namely the globular equiaxed crystal. One may argue that the real Fe–C–Mn alloy system tends to solidify in a columnar or mixed columnar-equiaxed structure, but for evaluating the model we consider here only equiaxed solidification. Simulation of mixed columnar-equiaxed solidification is presented in Part 2. A 2D square casting (50 × 50 mm^2^) is meshed into volume elements of 2.5 × 2.5 mm^2^. The casting starts to solidify from a uniform initial temperature *T*_0_. Both the die temperature, $T_{w}$, and the heat transfer coefficient at the casting–die interface, $H_{w}$, are set as constant. The material properties and other parameters used for the simulations are summarized in Table 2. Different simulation cases were defined (Table 3). The calculations are performed transiently in 2D, but the analysis of the solidification path is performed at specified points, e.g. at the casting center and at one of the corners.

**Table 2:** Material properties and other parameters used for the simulations.

*Thermophysical properties*				
Specific heat	*c_p_*_(_*_ℓ_*_)_, *c_p_*_(_*_s_*_)_	500	J kg^−1^ K^−1^	Ref. [39]
Diffusion coeff.	*D_ℓ_*_,C_	2 × 10^−8^	m^2^ s^−1^	[30]
	*D_ℓ_*_,Mn_	4 × 10^−9^	m^2^ s^−1^	[34], [35]
	*D_s_*_,C_	1 × 10^−9^	m^2^ s^−1^	[30]
	*D_s_*_,Mn_	1.2 × 10^−13^	m^2^ s^−1^	[21]
Latent heat	Δ*h_f_*	2.71 × 10^5^	J kg^−1^	[39]
Heat conductivity	*k_ℓ_*, *k_e_*, *k_c_*	34	W m^−1^ K^−1^	[39]
Thermal exp. coeff.	*β_T_*	1.43 × 10^−4^	K^−1^	[36]
Solutal exp. coeff.	*β_c_*_,C_	1.1 × 10^−2^	wt.%^−1^	[21]
	*β*_c,Mn_	0.2 × 10^−2^	wt.%^−1^	[21]
Density	*ρ_ℓ_*, *ρ_e_*, *ρ_c_*	6990	kg m^−3^	[39]
Boussinesq density diff.	Δ*ρ*	150	kg m^−3^	[34]
Viscosity	*μ*	4.2 × 10^−3^	kg m^−1^ s^−1^	[39]

*Thermodynamic parameters*				
Partition coeff.	*k*_C_	0.36	–	[36]
	*k*_Mn_	0.75	–	[21]
Liquidus slope	*m*_L,C_	−55	K/wt.%	[38]
	*m*_L,Mn_	−4.8	K/wt.%	[38]
Eutectic temp.	*T_E_*	1426.15	K	[16]
Melting point of Fe	*T_f_*	1805.15	K	[16]
Gibbs–Thomson coeff.	*Γ*	1.9 × 10^−7^	m K	[30]
Primary DAS	$\lambda_{1}$	5 × 10^−4^	m	[39]

*Process parameters (I.C. and B.C.)*				
Initial temp.	*T*_0_	1777	K	
Heat transfer coeff.	*H_w_*	300	K	
Ambient temp.	*T_w_*	373	K	

*Nucleation parameters*				
Max. equiaxed number density	*n*_max_	2. × 10^9^	m^−3^	[16], [17]
Undercooling for max. nucl. rate	Δ*T_N_*	5	K	[16], [17]
Gaussian distribution width	Δ*T_σ_*	2	K	[16], [17]

*Others*				
Volume element	Δ*V*	1	mm^3^	
Time step	Δ*t*	0.001	s	
Vol. heat transfer between phases	*H*^∗^	10^−9^	W m^−3^ K^−1^	[16], [17]
Packing limit	$f_{e}^{c}$	0.637	–	[15]
Entrapment criterion	$f_{c}^{\text{free}}$	0.2	–	[16], [17]
CET blocking criterion	$f_{e}^{\text{CET}}$	0.49		[37]

**Table 3:** Definition of the simulation cases.

	Diff. coeff.^a^ (m^2^ s^−1^)	Comments
Case I	*D_ℓ_*_,_*_i_* = 10^−6^	Reproduce the analytical solution of lever rule, Eq. (11) with $a_{\text{C}}^{\prime }=a_{\text{Mn}}^{\prime }=0.5$
	*D_s_*_,_*_i_* = 10^−6^	
Case II	*D_ℓ_*_,_*_i_* = 10^−6^	Reproduce the analytical solution of Gulliver–Scheil, Eq. (11) with $a_{\text{C}}^{\prime }=a_{\text{Mn}}^{\prime }=0$
	*D_s_*_,_*_i_* = 0	
Case III	*D_ℓ_*_,_*_i_* = 10^−6^	Reproduce the analytical solution of finite back diffusion, Eq. (11) with following $a_{i}^{\prime }$^b^:
	*D_s_*_,_*_i_*: see Table 1	
		for a high cooling rate, $a_{\text{C}}^{\prime }=0.3941$, $a_{\text{Mn}}^{\prime }=0.00023$
		for a low cooling rate, $a_{\text{C}}^{\prime }=0.4165$, $a_{\text{Mn}}^{\prime }=0.000307$
Case IV	See Table 1	Full diffusion solidification kinetics where $c_{\ell \text{,}i}^{*}\neq c_{\ell \text{,}i}$ drives further growth/melting
Case V	See Table 1	Full diffusion solidification kinetics. A modified Case V, using the reduced liquid diffusion length 0.1 × *R_e_*(1 − *R_e_*/*R_f_*)

a Using a large diffusion coefficient (10^−6^) to reproduce the case of ‘infinite’ diffusion.

b $a_{i}^{\prime }$ is estimated according to the characteristic solidification time (*t_f_*) and solid back diffusion length (*l_s_*). Two different cooling rates are evaluated: one is at the corner of the square casting with a relatively short solidification time, $t_{f}$ = 30 s; one is at the casting center with a relatively long solidification time, $t_{f}$ = 40 s. The characteristic solid back diffusion lengths of both cases assume half of the average grain radius (0.25 mm).

### 3.1 Cases of infinite liquid mixing

**Fig. 4:**
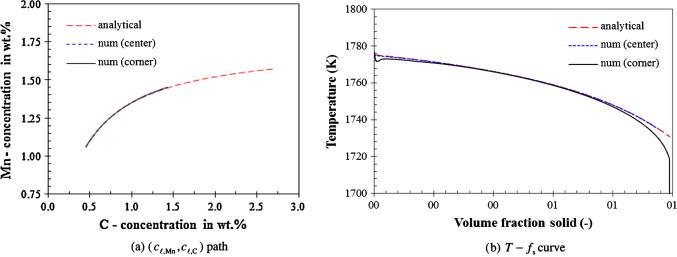
Solidification path of Case I (lever rule). The calculated paths of (*c_ℓ_*_,Mn_, *c_ℓ_*_,C_) at the casting corner and at the center overlie each other, while the $T-f_{s}$ curves show the difference from each other at the early stage and end of solidification.

**Fig. 5:**
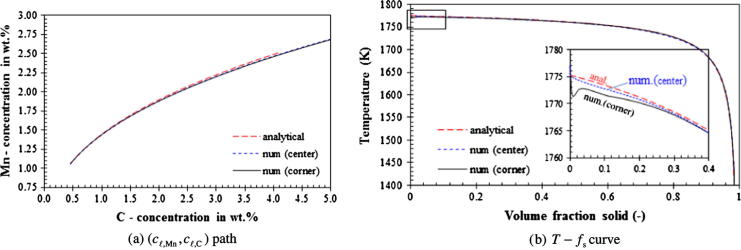
Solidification path of Case II (Gulliver–Scheil). The calculated (*c_ℓ_*_,Mn_, *c_ℓ_*_,C_) paths at the casting corner and center overlie each other, while the $T-f_{s}$ curves show the difference from each other at the early stage of solidification.

**Fig. 6:**
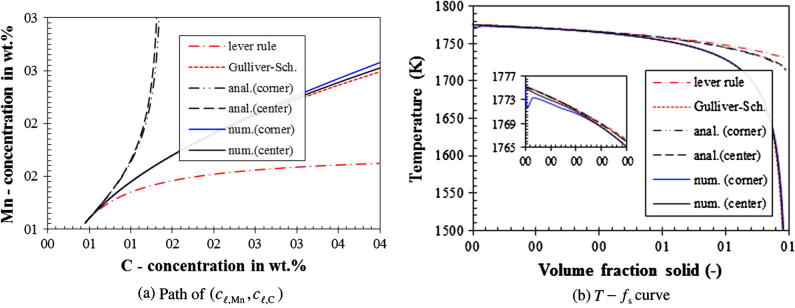
Solidification path of Case III (finite back-diffusion). The numerically calculated path of (*c_ℓ_*_,Mn_, *c_ℓ_*_,C_) at the casting corner is almost identical to that at the casting center, while the analytical paths of the two points show differences. For the sake of comparison, the lever rule and Gulliver–Scheil results are plotted as well.

The numerical results of the solidification path for the three ‘infinite’ liquid mixing cases, Case I–III, are shown in Fig. 4, Fig. 5, Fig. 6 and compared with the analytical solutions. A very large liquid diffusion coefficient, 10^−6^ m^2^ s^−1^, is used to mimic the ‘infinite’ liquid mixing in the numerical model. From our results it can be observed that the numerically calculated $c_{\ell \text{,}i}^{*}$ is almost equal to $c_{\ell \text{,}i}$. Therefore, only the path of (*c_ℓ_*_,Mn_, *c_ℓ_*_,C_) is necessarily evaluated.

For Case I (lever rule), a very large solid diffusion coefficient, 10^−6^ m^2^ s^−1^, is also assumed in the numerical model. As expected, the numerically calculated (*c_ℓ_*_,Mn_, *c_ℓ_*_,C_) path agrees with the analytical solution. The calculated $T-f_{s}$ curve agrees to a great extent with the analytical solution. Differences are found for the corner point at the very beginning and at the end of the solidification. Although a very large diffusion coefficient is assumed for both liquid and solid phases, the calculated diffusion rate is still not high enough to be comparable with the ideal lever rule assumption (infinite diffusion in solid phase and perfect mixing in liquid). An additional reason for the delay of solidification at the beginning is due to the nucleation. As can be seen in Fig. 4b, this discrepancy becomes insignificant with the slower cooling rate at the casting center.

For Case II (Gulliver–Scheil), both numerical and analytical (*c_ℓ_*_,Mn_, *c_ℓ_*_,C_) paths agree with each other (see Fig. 5). Different cooling rates at the casting corner and the casting center have no influence on the (*c_ℓ_*_,Mn_, *c_ℓ_*_,C_) paths. The same is true for the $T-f_{s}$ curves estimated at the two points. Only a minor difference is observed at the very beginning of solidification due to the nucleation.

For Case III (finite back diffusion), it is difficult to obtain an agreement between numerical and analytical results (see Fig. 6). The reason is that the analytical solution is based on an estimated constant solid diffusion length (0.25 mm), which takes half of the average grain radius of the finally as-solidified structure. In contrast, the numerical simulation takes $R_{e}$/2 as the solid diffusion length, which evidently varies during solidification. The numerical result of the (*c_ℓ_*_,Mn_, *c_ℓ_*_,__C_) path and $T-f_{s}$ curve seems closer to the Gulliver–Scheil results rather than they do to the analytical ones given by Eqs. (11), (12).

### 3.2 Cases of full diffusion-governed solidification kinetics

**Fig. 7:**
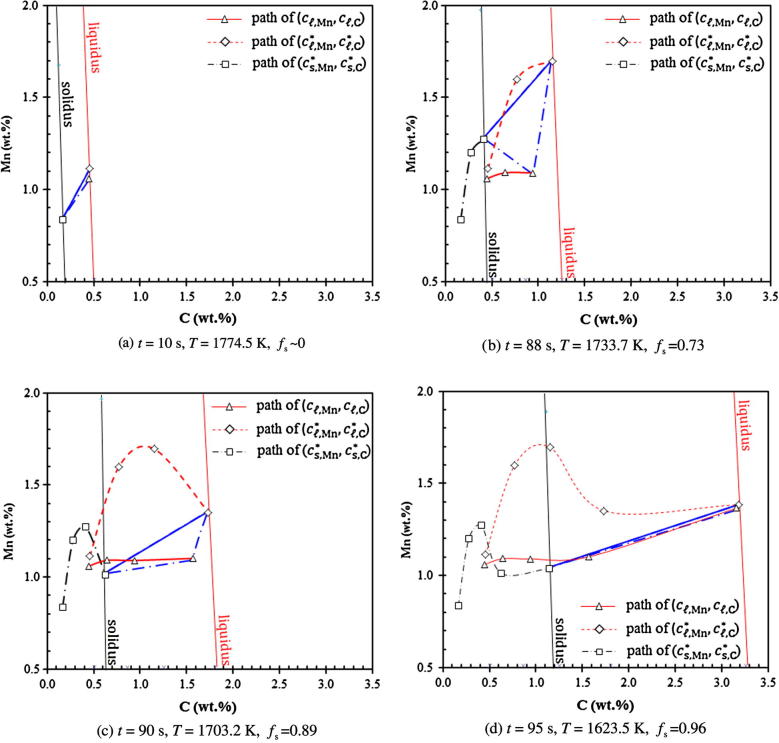
Predicted solidification path of Case IV (evaluated at the casting center): paths of (*c_ℓ_*_,Mn_, *c_ℓ_*_,C_), ($c_{\ell \text{,Mn}}^{*}\text{,}c_{\ell \text{,C}}^{*}$) and ($c_{s\text{,Mn}}^{*}\text{,}c_{s\text{,C}}^{*}$). Tie-lines at different times are plotted. Liquidus and solidus isolines are also plotted at the corresponding temperatures.

**Fig. 8:**
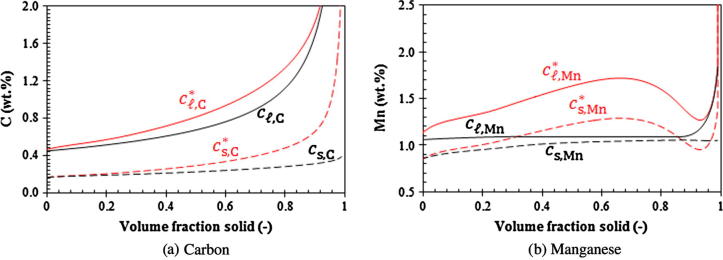
Calculated concentrations as a function of volume fraction solid (Case IV).

For the case of full diffusion-governed solidification (Case IV), where we allow $c_{\ell \text{,}i}^{*}$ to differ from $c_{\ell \text{,}i}$, the resulting solidification path is evaluated by the (*c_ℓ_*_,Mn_, *c_ℓ_*_,C_), ($c_{\ell \text{,Mn}}^{*}\text{,}c_{\ell \text{,C}}^{*}$) and ($c_{\text{s,Mn}}^{*}\text{,}c_{\text{s,C}}^{*}$) paths (see Fig 7). The calculated concentrations as a function of volume fraction solid are shown in Fig. 8.

Before solidification starts, the volume-averaged concentrations (*c_ℓ_*_,C_, $c_{\ell \text{,Mn}}$) remain constant (initial composition of the alloy). As soon as the solidification starts, both the (*c_ℓ_*_,Mn_, *c_ℓ_*_,C_) and ($c_{\ell \text{,Mn}}^{*}\text{,}c_{\ell \text{,C}}^{*}$) paths starts to depart from each other (Fig. 7). The ($c_{\ell \text{,Mn}}^{*}\text{,}c_{\ell \text{,C}}^{*}$) path bends upwards while the (*c_ℓ_*_,Mn_, *c_ℓ_*_,C_) path responses much slowly. The volume-averaged liquid Mn-concentration, $c_{\ell \text{,Mn}}$, increases slightly at the initial stage until $f_{s}$ reaches ca. 0.31. Then it remains almost constant, while the volume-averaged liquid C-concentration, $c_{\ell \text{,C}}$, continues to increase (see Fig. 8). As $k_{\text{C}}$ and $k_{\text{Mn}}$ are smaller than 1, solute partitioning for both alloy elements occur and the volume-averaged liquid concentrations (*c_ℓ_*_,C_,*c_ℓ_*_,Mn_) are initially enriched. The enrichment rate of both $c_{\ell \text{,C}}$ and $c_{\ell \text{,Mn}}$ is much smaller than the increase rate of equilibrium concentrations at the interface ($c_{\ell \text{,Mn}}^{*}\text{,}c_{\ell \text{,C}}^{*}$) and ($c_{s\text{,Mn}}^{*}\text{,}c_{s\text{,C}}^{*}$) due to the rapid drop in temperature. In accordance with the current model, the species transfer due to solidification is determined by the mass transfer interfacial concentration, $c_{s\text{,}i}^{\text{Intf}}$. Two solute partition mechanisms are considered. For the first mechanism when $c_{s\text{,}i}^{*}$ is smaller than $c_{\ell \text{,}i}$, $c_{s\text{,}i}^{\text{Intf}}$ is taken to be $c_{s\text{,}i}^{*}$. The solute partitioning between the liquid and solid causes enrichment of the solute in the liquid. For the second mechanism when $c_{s\text{,}i}^{*}$ becomes larger than $c_{\ell \text{,}i}$, $c_{s\text{,}i}^{\text{Intf}}$ is taken to be $c_{\ell \text{,}i}$, and no solute enrichment would occur. The second case does happen to Mn. Mn has a relatively large partition coefficient (0.75, closer to 1) and a relatively small diffusion coefficient in liquid. As seen in Fig. 8b, at the moment when *f_s_* reaches 0.31, $c_{s\text{,Mn}}^{*}$ becomes larger than $c_{\ell \text{,Mn}}$. Afterwards, there is no longer any enrichment of $c_{\ell \text{,Mn}}$. In fact, *c_ℓ_*_,Mn_ decreases slightly due to back diffusion.

At the late stage of solidification, the solute field impingement between neighboring grains causes the rapid reduction of the liquid diffusion length (*l_ℓ_*_,_*_e_* = *R_e_*(1 − *R_e_*/*R_f_*)). This brings the paths of (*c_ℓ_*_,Mn_, *c_ℓ_*_,C_) and ($c_{\ell \text{,Mn}}^{*}\text{,}c_{\ell \text{,C}}^{*}$) together again (Fig. 7). The equilibrium concentration of Mn, $c_{\ell \text{,Mn}}^{*}$, is ‘drawn’ close to the volume-averaged concentration, $c_{\ell \text{,Mn}}$. At the moment when $c_{s\text{,Mn}}^{*}$ again becomes smaller than $c_{\ell \text{,Mn}}$ (*f_s_* = 0.86, Fig. 8b), the solute partition is ‘switched’ back to the solute partition mechanism of ‘$c_{s\text{,}i}^{\text{Intf}}=c_{s\text{,}i}^{*}$’. The enrichment of Mn continues.

Imaginarily, if the solute field impingement between neighboring grains were ignored, i.e. assuming $l_{\ell \text{,}e}=R_{e}$ (estimated diffusion length for an spherical crystal growing in an infinite volume of liquid melt), the two paths of (*c_ℓ_*_,Mn_, *c_ℓ_*_,C_) and ($c_{\ell \text{,Mn}}^{*}\text{,}c_{\ell \text{,C}}^{*}$) would continue to depart from each other and never come together at the end stage of solidification. It would lead to an error prediction of the solidification path at the end stage of solidification.

**Fig. 9:**
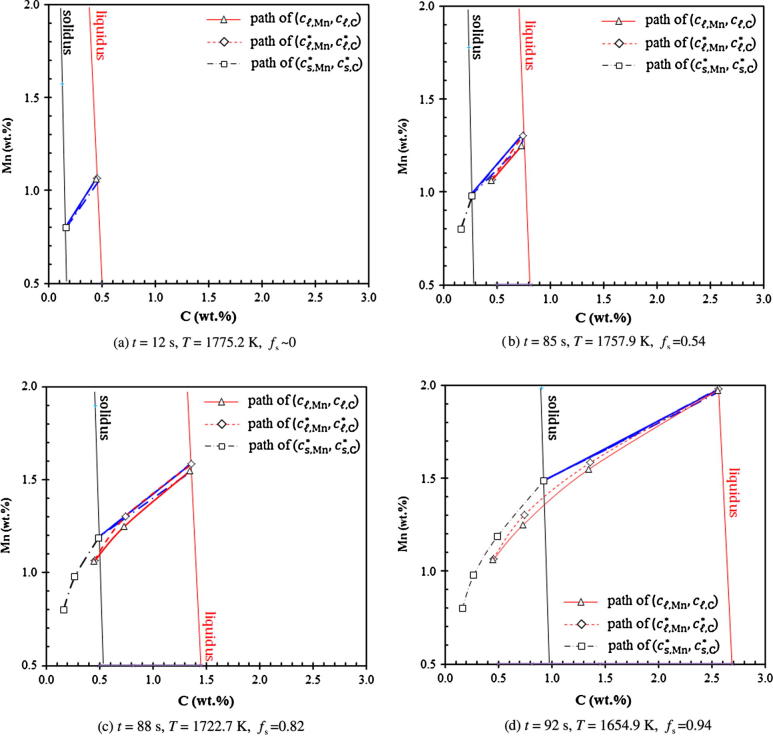
Predicted solidification path of Case V (evaluated at the casting center). Case V is modified by reducing the liquid diffusion length by a factor of 10 (*l_ℓ_*_,_*_e_* = 0.1 × *R_e_*(1 − *R_e_*/*R_f_*)). Liquidus and solidus isolines are also plotted at the corresponding temperatures.

**Fig. 10:**
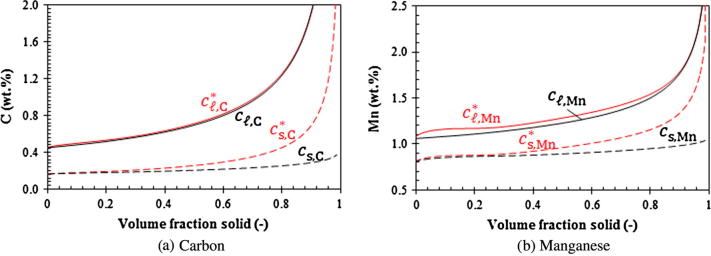
Calculated concentrations as a function of volume fraction solid (Case V).

The above calculations were based on the globular equiaxed solidification. Most technical alloys solidify in dendritic morphology. The estimated diffusion lengths in Table 1 apply well to the initial stage of solidification when the crystals are globular, but they do not apply to the dendritic solidification. For dendritic solidification alternative methods should be used to calculate the diffusion lengths [40], [41], [42], [43], [44], [45], [46], [47], [48]. In the case of dendritic solidification, the diffusion length is usually in the magnitude of the secondary dendrite arm spacing ($\lambda_{2}$) [25], [26] which is approximately one order of magnitude smaller than the grain size (*R*_e_). An additional simulation (Case V) was performed using a reduced diffusion length, 0.1 × *R_e_*(1 − *R_e_*/*R_f_*). The results are shown in Fig. 9, Fig. 10. Due to the reduced diffusion length, the solute ‘mixing’ in the liquid is significantly enhanced and the difference between $c_{\ell \text{,}i}$ and $c_{\ell \text{,}i}^{*}$ is dramatically reduced. Both Mn and C are gradually enriched with solidification. In comparison to Case IV, the solidification path calculated by Case V becomes much closer to the infinite liquid mixing approaches (Case I–III).

## 4 Discussion and conclusion

The aim of the present work is to develop and evaluate a multiphase process-scale solidification model incorporating the full diffusion-governed solidification kinetics and ternary phase diagram with the macroscopic transport phenomena. A key feature of this model, distinguishing it from the previous models [5], [6], [7], [8], [9], [10], [30], [32], [33], is that the full diffusion-governed solidification kinetics is considered for the solidification of a ternary alloy system. The importance of considering the liquid diffusion was demonstrated with the case simulations of a ternary alloy (Fe–0.45 wt.%C–1.06 wt.%Mn). Analytical solutions of several special cases with assumptions of lever rule or Gulliver–Scheil were reproduced by the current model, using the ‘infinite’ liquid diffusivity and the ‘infinite’ or ‘0’ solid diffusivity.

With the finite liquid diffusion, the volume-averaged liquid concentrations (*c_ℓ_*_,C_, *c_ℓ_*_,Mn_) are predicted to differ significantly from the equilibrium concentrations ($c_{\ell \text{,C}}^{*}\text{,}c_{\ell \text{,Mn}}^{*}$). The enrichment of $c_{\ell \text{,C}}$ and *c_ℓ_*_,Mn_ is caused by the solute partition at the solid–liquid interface, and the diffusion into the liquid. Therefore, the solidification path depends strongly on the solute diffusivity, the diffusion length, and the partition coefficient. The predicted solidification path of the considered ternary alloy differs significantly from those calculated by the classical models of infinite liquid mixing (e.g. lever rule, Gulliver–Scheil, or the cases of limiting solid back diffusion). The chosen element Mn has a relatively small diffusion coefficient and large partition coefficient, while the enrichment of $c_{\ell \text{,Mn}}$ is very small. During the solidification, the solidus concentration, $c_{s\text{,Mn}}^{*}$, may become even larger than $c_{\ell \text{,Mn}}$ and thus a simplified solute trapping mechanism ($c_{s\text{,}i}^{\text{Intf}}=c_{\ell \text{,}i}$) is considered. The solute element Mn in liquid is assumed to be fully ‘trapped’ in the solid. The enrichment of $c_{\ell \text{,Mn}}$ would not continue in the subsequent solidification process. This treatment is crude. Further improvement is desired to consider a transition from the equilibrium solute partitioning to a full solute trapping. However, this numerical treatment supports one general experimental fact: the segregation phenomenon disappears in a low-diffusive alloy under rapid solidification.

Evidently, the appropriate estimation of the diffusion length becomes an important issue for determining the diffusion-governed solidification path. The current model provides reasonable result at the initial stage of solidification, but it might cause erroneous estimation at the late stage of solidification. Although the solute field impingement at the end stage of solidification is considered, the evolution of dendritic morphology is not treated. For the case of dendritic solidification alternative methods should be used [40], [41], [42], [43], [44], [45], [46], [47], [48] to calculate the diffusion length.

Current paper used the locally linearized phase diagram. It is not difficult to extend this work for a more general case with precise thermodynamic information. In fact, one way to access the thermodynamic data (Thermo-Calc) by means of a tabulation and interpolation program ISAT (In Situ Adaptive Tabulation) was suggested by the authors [23], [24]. However, the calculation cost is extremely high. With the future development of computation resources, this method should be used.

The diffusion-governed solidification of a ternary alloy with flow and grain sedimentation, and the diffusion kinetics on macrosegregation are investigated in the second part of this two-part investigation [Part 2].

## Appendix A.

Solution procedure of the non-linear equation system, Eqs. (7), (8), (9), (10):

Rewriting Eq. (7) in the form:

(A.1) $$a=b\cdot c_{\ell \text{,}A}^{*}+c\cdot c_{\ell \text{,}B}^{*}$$

and inserting Eqs. (8), (9) into Eq. (10) gives:

(A.2) $$d\cdot c_{\ell \text{,}B}^{*}\left(c_{\ell \text{,}A}^{*}-c_{\ell \text{,}A}\right)+e\cdot c_{\ell \text{,}B}^{*}\left(k_{A}c_{\ell \text{,}A}^{*}-c_{s\text{,}A}\right)=f\cdot c_{\ell \text{,}A}^{*}\left(c_{\ell \text{,}B}^{*}-c_{\ell \text{,}B}\right)+g\cdot c_{\ell \text{,}A}^{*}\left(k_{B}c_{\ell \text{,}B}^{*}-c_{s\text{,}B}\right)$$

where

$$\left\{\begin{array}{c} a=T-T_{f}\\ b=m_{L\text{,}A}\\ c=m_{L\text{,}B}\\ d=(1-k_{B})\cdot D_{\ell \text{,}A}/l_{\ell \text{,}c}\\ e=(1-k_{B})\cdot D_{s\text{,}A}/l_{s\text{,}c}\\ f=(1-k_{A})\cdot D_{\ell \text{,}B}/l_{\ell \text{,}c}\\ g=(1-k_{A})\cdot D_{s\text{,}B}/l_{s\text{,}c} \end{array}\right)$$

By further combining Eqs. (A.1), (A.2), a parabolic equation for $c_{\ell \text{,}A}^{*}$ is obtained,

(A.3) $$A\cdot c_{\ell \text{,}A}^{*2}+B\cdot c_{\ell \text{,}A}^{*}+C=0$$

with

$$\left\{\begin{array}{c} A=\mathrm{bf}+\mathrm{bg}\cdot k_{B}-\mathrm{bd}-\mathrm{be}\cdot k_{A}\\ B=\mathrm{ad}+\mathrm{bd}\cdot c_{\ell \text{,}A}+\mathrm{ae}\cdot k_{A}+\mathrm{be}\cdot c_{s\text{,}A}-\mathrm{af}+\mathrm{cf}\cdot c_{\ell \text{,}B}-\mathrm{ag}\cdot k_{B}+\mathrm{cg}\cdot c_{s\text{,}B}\\ C=-\mathrm{ad}\cdot c_{\ell \text{,}A}-\mathrm{ae}\cdot c_{s\text{,}A} \end{array}\right)$$

The solution of Eq. (A.3) becomes apparent. The Newton–Raphson method is employed to solve Eq. (A.3) iteratively. The solution $c_{\ell \text{,}A}^{*}$ of the last time step is used as an initial value for the next time step. As $c_{\ell \text{,}A}^{*}$ is known, one can subsequently obtain $c_{\ell \text{,}B}^{*}$ from Eq. (A.1), $c_{s\text{,}A}^{*}$ and $c_{s\text{,}A}^{*}$ from Eqs. (8), (9). Please note that the above symbols *a* through *g*, and *A* through *C* apply only in this appendix.
